# Internet Addiction

**DOI:** 10.3390/life12060861

**Published:** 2022-06-09

**Authors:** Fehér Gergely

**Affiliations:** Centre for Occupational Medicine, Medical School, University of Pecs, 7624 Pecs, Hungary; feher.gergely@pte.hu

This topic was dedicated to the complexity of internet addiction and contains 23 articles submitted by prestigious workgroups and originally launched as a Special Issue entitled “Internet Addiction”. Due to the large number of manuscripts and the diversified approaches, these papers were originally published by four high-quality MDPI journals. I hope this set of articles can help us in a more accurate understanding of the development of compulsive internet use.

The extensive availability of the internet has led to the recognition of problematic internet use (so-called internet addiction, IA). Problematic internet use is usually defined as a problematic, compulsive use of the internet, resulting in significant impairment in an individual’s function in various life domains over a prolonged period of time. This disorder is increasingly prevalent; about 5% of the whole population is considered to suffer from it. A difficulty in recognizing this disorder is that internet-based technology has improved many aspects of our lives, and it is now an essential part of our everyday routine, including work, private life, and social functioning; therefore, many individuals are not aware of the misuse or problematic use [1].

IA seems to have several risk factors. The most important ones are a younger age at the start of internet use and being male [2]. An extensive Czech study conducted by Kopecky et al. including more than 27,000 students aged between 7 and 17 years showed that they spent a large part of their leisure time at school using their mobile phones linked to one of the social networks, YouTube, and videogames instead of practicing sports or social activities [3]. In the absence of their digital devices, they had to face boredom and loneliness, which underlines the importance of proper control [3]. Furthermore, the unlimited and exaggerated use of mobiles devices/phones may be associated with a new psychiatric term entitled nomophobia (the acronym means “no-mobile-phobia”), described as experiencing intense fear anxiety, stress, and discomfort due to the idea of being without a mobile phone or the inability to use it [4]. A systematic review and meta-analysis conducted by Humood and his colleagues showed that the prevalence of severe nomophobia can be as high as approximately 21% in the general population and about 9% in high school students [5]. However, in their meta-regression analysis, neither age nor sex was a significant predictor, so these results merit further investigation, but also underline the importance of digital control.

Family functioning also has a crucial role in the development of IA [6]. The study published by Li et al. showed that better-functioning families are less likely to be associated with problematic internet use [7]. Kapus and his workgroup also showed the negative role of impaired family relationships such as living without parents was a significant predictor of IA in their extremely impressive study, which took many co-variates into account [8]. 

One’s place of stay may have an influence of IA, as problematic use seems to be more common in rural areas and among those with low socioeconomic status [9]. In their interesting research paper, Yasuma et al. retrospectively analyzed the role of urban upbringing on IA and found a positive association even after adjusting for both sociodemographic characteristics and psychopathology (including measuring psychological distress and a history of mental illnesses in the past 12 months) [10]. 

Certain individual personality traits appear to be common among adolescents with problematic internet use [2,11]. According to the paper by Li et al., adolescents with high self-control showed a high level of consideration of future outcomes, which in turn reduced internet addiction [12]. Among a sample of Spanish university students, some social skills variables such as conversation and social ease, empathic and positive feeling skills, and risk coping predicted problematic internet use in a multivariate analysis, and Romeró-Lopez and his colleagues also found the increased risk among younger students having obsessive thoughts about being online and having a higher risk of IA [13]. Zeng et al.’s research focused on the possible association and pathways among self-esteem, individual affect, relationship satisfaction, and IA. Mediation analysis of their sample indicated that negative affect and relationship dissatisfaction mediated the relationship between self-esteem and IA [14]. A high tendency toward novelty seeking was previously shown to be associated with IA, and Lane and his workgroup also found that it was associated with smartphone addiction [2,11,15]. 

Apart from the time spent online (spending more and more time online is a tentative indicator of tolerance, the core criterion of dependence), certain online activities are deliberately made to be addictive, such as online gaming [16]. Although internet addiction is not labelled as a medical condition, gaming disorder was included both in the appendix of the DSM-V (as a potential warning condition) and in the ICD-11 as a medical condition [17,18]. Despite warning signs, competitive online video gaming (or esports) is extremely prevalent in our century and is considered a healthy hobby for the vast majority of people (also promoted as such by developers) [19]. About 1 out of 5 recreational adult esports players can be compulsive internet users, which is significantly higher than the estimated 5% prevalence according to the paper by Kósa et al., and this study also draws attention to the risk factors for IA, such as younger age, family status, and type of employment, and its possible association with depression and alcohol use (abuse) [20]. Watching videos, including live streams, can also be additive, as shown by a pilot interview study, but larger samples are needed to clarify the possible association [21]. 

Problematic internet use seems to be associated with several mental and medical conditions [22,23]. According to a Russian study conducted by Tereshchenko et al., problematic internet users struggle with significant disturbances in the quality of nighttime sleep and excessive daytime sleepiness [24]. Depressive symptoms predicted internet addiction but not vice versa at a within-person level in a Chinese study [25]. Yi et al. also found male predominance and suggested effective identification and intervention of depressive symptoms in the intervention and prevention of internet addiction [25]. Kożybska and his colleagues showed the possible association of IA and eating disorders [26]. Significant predictors were the preoccupation with the Internet, neglect of sleep in favor of online activity, relieving negative feelings while online, and a higher average number of weekends hours spent online on study/work-related activities, extracurricular activities (working, active membership in student organizations), lower height, and higher body mass index [26]. 

Similar to other addictions, the withdrawal from internet use can be associated with severe symptoms, with a disruption of the brain nerve networks that implement both time control and autonomic nervous regulation of cardiac activity based on a Russian study carried out by Krivonogova et al. [27]. 

The recently recognized coronavirus disease (COVID-19) and pandemic, starting in 2019 with subsequent lockdown, home quarantine, and homework/home education, has dramatically increased the use of web-based technologies [28]. Frequent and extensive uses of the internet were shown to be strong predictors of physical health problems by a Bangladesh study concluded by Abir et al. [29]. The time spent on internet gaming also significantly increased among adolescents based on a representative survey from South Korea by Kim et al. [30]. However, a subgroup analysis showed that the majority of adolescents did not exhibit significant aggravation of addictive internet gaming usage during the COVID-19 pandemic, except for compulsive gamers [30]. 

Problematic internet use is extremely well studied among adolescents, but far fewer research studies are available in adults [1].

Pohl et al. showed that the rate of IA can be as high as 5% among Hungarian teachers, and it can be associated with burnout, moderate and severe depression, insomnia, and lower quality of life in all domains [31]. In a multivariate analysis, internet addiction was shown to be significantly associated with depression and insomnia, which underlines the importance of this phenomenon [31]. In a large sample of Portuguese workers, IA was also strongly associated with anxiety and depression, as concluded by Pereira et al. [32]. Apart from problematic internet use, age (being older), gender (being female), not having enough economic funds, being unsatisfied with the leadership in the job, being unsatisfied with the nature of the job, and having higher scores in salience were significant predictors of mental health issues [32]. 

IA may be classified as a compulsive-impulsive spectrum disorder based on symptomatology, but it has been undergoing considerable research and is not included in the most recently published 5th edition of the Diagnostic and Statistical Manual DSM-V [33]. Masters et al. also showed in their systematic literature review that researchers do not control for academic work activity when measuring medical students’ internet addiction and that researchers should use more granular tools (as the currently used tools seem to be far too blunt) and specifically control for work-related activities [34]. 

Similar to internet addiction (which is not entirely clarified, as proper criteria and treatment modalities are missing), internet gaming disorder (IGD) has also become an important health concern in a significant proportion of adolescents, as seen above [1,33]. As randomized studies are missing, we face a lack of guidelines. A recent study raised the possible efficacy of several treatment modalities, namely psychoeducation, emotion management, behavior analysis and modification, social skills training, parent participation, and relapse prevention, which have been described to be effective by participants and experts [35]. 

Fear of missing out (FoMO) is a relatively new concept meaning the fear of missing satisfying experience when the individual is absent from 239 his/her companions and having a strong desire to always be with others [36]. It is strongly related to mental issues such as smartphone addiction, compulsive social media use, internet addiction, phubbing behavior, insomnia, and poorer academic performance [37]. This topic also contains the Chinese validation of this questionnaire, which may help to explore the psychopathology leading to internet addiction [37].

However, the widespread use of the internet has not only disadvantages but also a marked dampening effect. Xie et al. concluded in their extensive scientometric analysis that new media have almost brought about a new era for maternal health, mainly characterized by psychological qualities, healthy, and reasonable physical conditions and advanced technology [38].

Finally, our Special Issue was surrounded by a great deal of interest, which is reflected in the large number of manuscripts submitted and published. We are pleased to announce that, in view of the above, the topic will be republished, and details will soon be published on our website.

I hope you can enjoy this brilliant research.
